# Supplementary material for: Validation and repurposing of the MSL-COVID-19 score for prediction of severe COVID-19 using simple clinical predictors in a triage setting: The Nutri-CoV score
Source: PLoS One. 2020 Dec 16;15(12):e0244051. doi: 10.1371/journal.pone.0244051 (PMC7743927; doi:10.1371/journal.pone.0244051)

**S1 File.**

**Supplementary Table 1.** Results from model selection after multiple imputation using the LASSO procedure with the *MAMI* R package. Variables which had a coefficient equal to zero in Fit 1 were removed from the model and refitted (Fit 2).

| Fit 1 | | |
| --- | --- | --- |
| Variable | Estimate | Std.Error |
| Sex | 0.07317252 | 0.12872092 |
| Fever | 0 | 0.11985199 |
| Cough | 0 | 0.12673311 |
| Headache | -0.1152623 | 0.12331556 |
| Dyspnea | 0.01683464 | 0.12555296 |
| Irritability | 0.13817823 | 0.14698172 |
| Diarrhea | 0 | 0.07978259 |
| Chest pain | -0.1181772 | 0.1487667 |
| Shivers | -0.0197343 | 0.0924933 |
| Odynophagia | 0 | 0.08125264 |
| Myalgias | -0.0844104 | 0.12895159 |
| Arthalgias | -0.0080373 | 0.08671487 |
| General Malaïse | 0 | 0.11113469 |
| Rinorrhea | 0 | 0.07563272 |
| Vomiting | 0 | 0.10893817 |
| Abdominal pain | 0.01800659 | 0.14549773 |
| Conjuntivitis | 0 | 0.10910196 |
| Cyanosis | 0.00426571 | 0.16504278 |
| Temperature | 0.02113993 | 0.06893824 |
| Heart rate | 0 | 0.00271755 |
| Respiratory rate | 0.0121487 | 0.0086335 |
| SpO_2_ | -0.0261711 | 0.005423 |
| SBP | -1.63E-05 | 0.00244367 |
| DBP | -0.0044474 | 0.0052642 |
| MSL-COVID-19 | 0.01765379 | 0.01552729 |
| Supplementary O_2_ | 0.00983672 | 0.08359407 |
| Fit 2 | | |
| Variable | Estimate | 95%CI |
| Sex | 0.02347632 | (-0.203, 0.250) |
| Headache | -0.1646632 | (-0.424, 0.094) |
| Dyspnea | 0 | (-0.171, 0.171) |
| Irritability | 0.19943649 | (-0.151, 0.549) |
| Chest Pain | -0.1401806 | (-0.496, 0.215) |
| Shivers | -0.0369917 | (-0.266, 0.192) |
| Myalgias | -0.1051116 | (-0.396, 0.186) |
| Arthalgias | 0 | (-0.184, 0.184) |
| Abdominal pain | 0.04347742 | (-0.313, 0.400) |
| Cyanosis | 0.02017096 | (-0.325, 0.365) |
| Temperature | 0.02225901 | (-0.129, 0.173) |
| **Respiratory rate** | **0.0101231** | **(0.007, 0.027)** |
| **SpO2** | **-0.0268868** | **(-0.037, -0.017)** |
| SBP | -0.0008492 | (-0.008, 0.006) |
| **MSL-COVID-19** | **0.04855388** | **(0.016, 0.081)** |
| O2 | 0.02475845 | (-0.181, 0.231) |

**Supplementary Table 2.** Comparison of k-fold cross-validation using k=10 and k=5 for the Nutri-CoV model. Elastic Net confidence intervals were estimated using simple bootstrapping within the training cohort and should be interpreted with caution. RR=Respiratory Rate; BPM= Breaths per minute; SpO2= Pulse oximetry.

| Parameter | k=5 | | | k=10 | | |
| --- | --- | --- | --- | --- | --- | --- |
|  | **β** | 95%CI | Points | **β** | 95%CI | points |
| MSL-COVID-19 risk category | 0.218 | 0.104, 0.331 | 1 | 0.219 | 0.105, 0.332 | 1 |
| RR 24-30bpm | 0.277 | -0.066, 0.620 | - | 0.2799 | -0.063, 0.621 | - |
| RR >30BPM | 0.478 | 0.129, 0.828 | 2 | 0.481 | 0.132, 0.829 | 2 |
| SpO2 85-92% | 1.212 | 0.121, 2.303 | 5 | 1.191 | 0.121, 2.261 | 5 |
| SpO2 <85% | 2.041 | 0.932, 3.149 | 9 | 2.018 | 0.923, 3.114 | 9 |

**Supplementary Figure 1.** Histogram and patterns of missing data in outcomes and candidate predictors within the evaluated cohorts. Data was multiply imputed using multivariable imputations with chained equations.


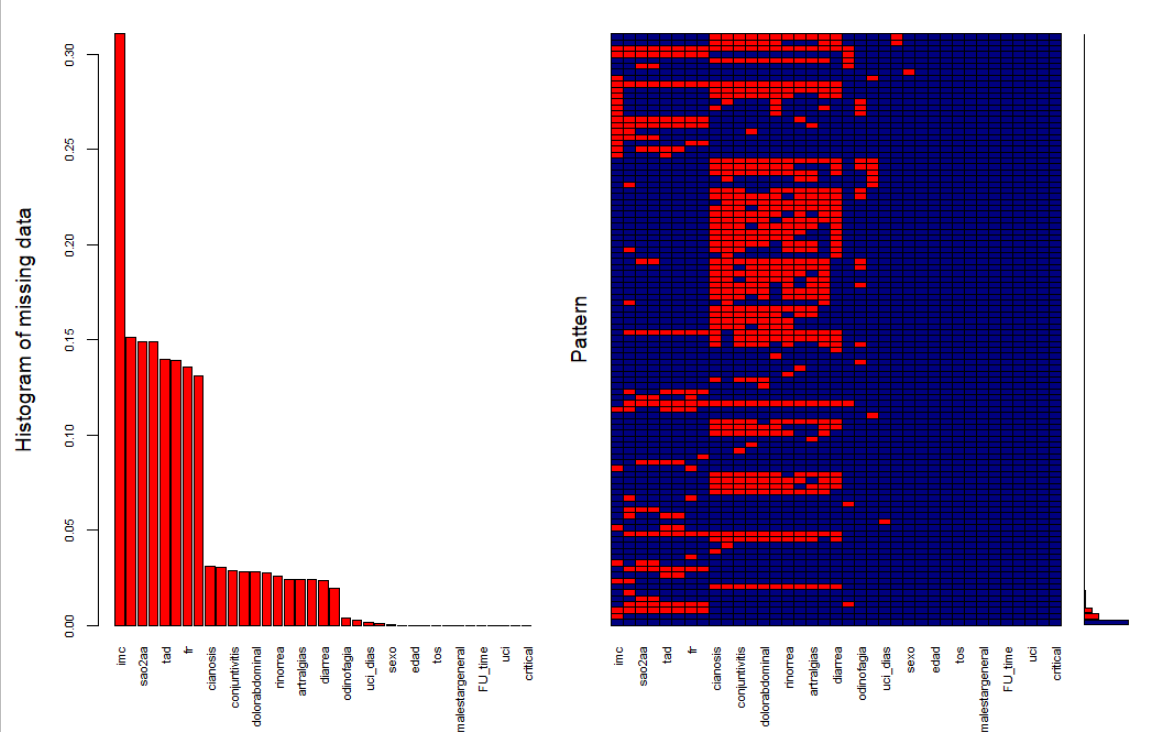


**Supplementary Figure 2.** Density plot of data prior (in red) and after multiple imputation (in blue) with five multiply imputed datasets.


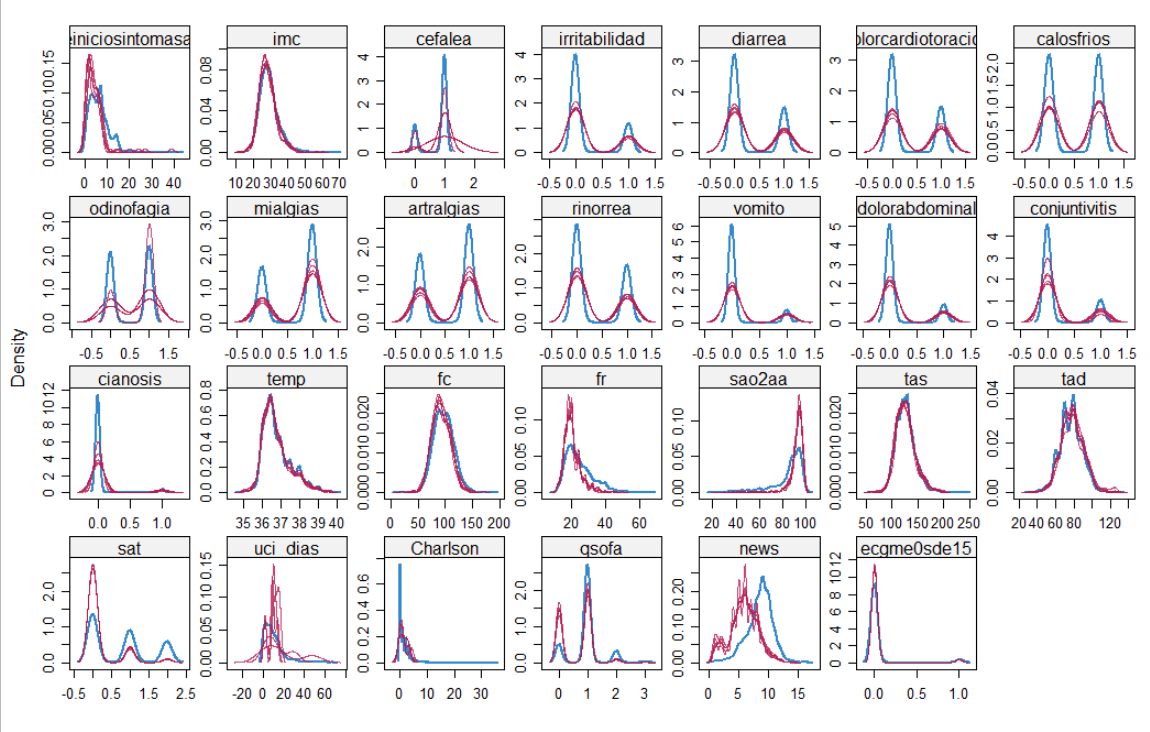


**Supplementary Figure 3.** Representation of Cox Proportional Hazard Regression models including Nutri-CoV components for pulse oximetry and respiratory rate modeled with non-linear terms using restricted cubic splines with 3 knots.


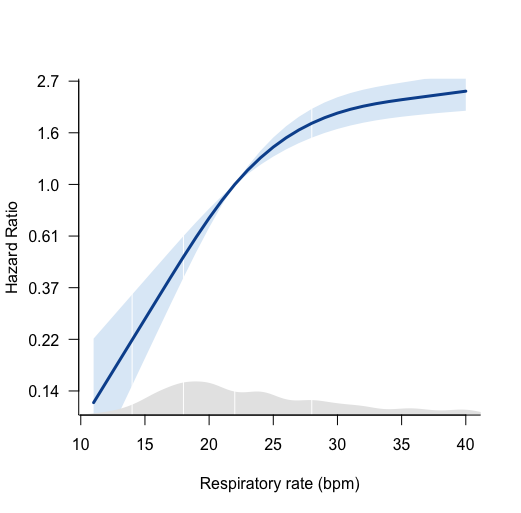

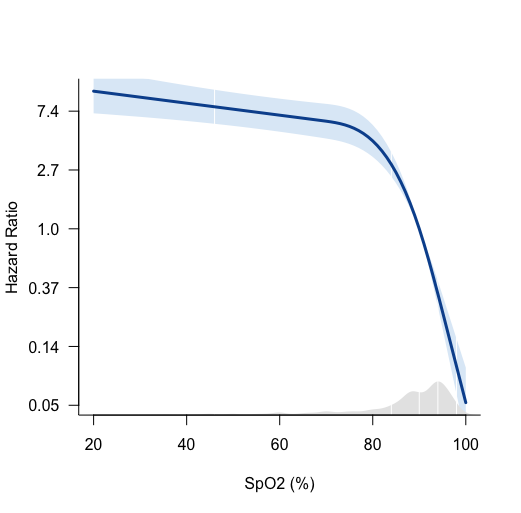


**Supplementary Figure 4.** Martingale residuals for MSL-COVID-19 validation

**
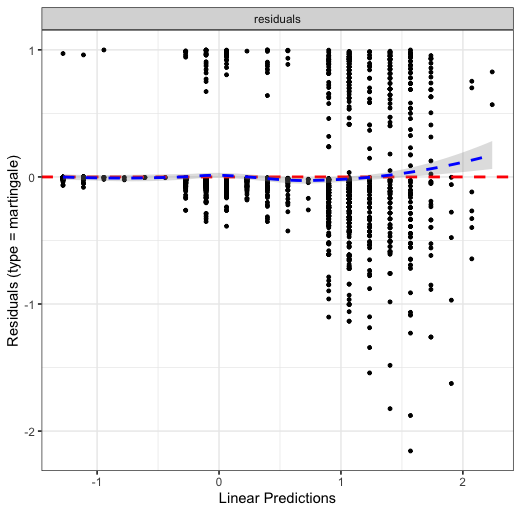
**

**Supplementary Figure 5.** Martingale and score residuals for the model comprising respiratory rate, MSL-COVID-19 and SpO2.


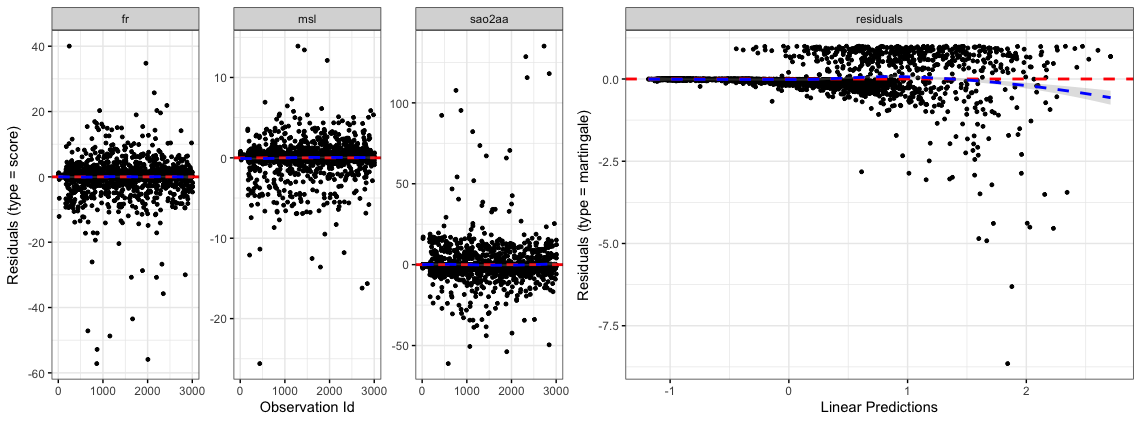

Supplement: S1 File — (DOCX) [file pone.0244051.s001.docx]
